# Seroprevalence of *Leptospira* in Lavradeiro horses, a semi-feral ecotype from the Northern Brazilian Amazon

**DOI:** 10.1007/s11259-026-11328-8

**Published:** 2026-06-09

**Authors:** Andressa da Silva Martins, Tamires Ataides Silva, Marcos Bryan Heinemann, Israel Barbosa Guedes, Rafael Rodrigues Soares, Ricardo Augusto Dias, Rebeca Farias Passos, Ramayana Menezes Braga, Fernanda Carlini Cunha dos Santos, Ana Carolina Borsanelli

**Affiliations:** 1 Departament of Preventive Veterinary Medicine, Universidade Federal de Goiás, Goiânia, Goiás Brazil; 2 Department of Preventive Veterinary Medicine and Animal Health, Universidade de São Paulo, São Paulo, Brazil; 3 Veterinary, Student at Graduate Program in Animal Reproduction in the Amazon (ReproAmazon), Pará, Brazil; 4 Embrapa Roraima, Boa Vista, Roraima Brazil; 5 Universidade Federal dos Vales do Jequitinhonha e Mucuri, Unaí, Minas Gerais Brazil

**Keywords:** Leptospirosis, Equine, Seroprevalence, Roraima, Tropical, Zoonosis

## Abstract

Leptospirosis is a neglected zoonosis with significant impact on public and veterinary health, with equines acting as potential maintenance hosts of infection. This study aimed to investigate the seroprevalence of *Leptospira* in Lavradeiro horses under semi-feral conditions in Indigenous communities in the state of Roraima, Brazil. A total of 387 serum samples were subjected to the microscopic agglutination test (MAT), using antigens of *Leptospira* spp. from 18 serogroups. The overall prevalence was 15.8% (95% CI: 12.3–19.8%), with the most frequent serogroups being Australis, Tarassovi, Autumnalis, and Pomona. Seropositivity was higher among adult animals (17.6%) than foals (9.8%), although not statistically significant. Between sexes, there was no significant difference in positive females (16.8%) and males (14.7%). Horses that lived in areas that were likely to flood had a higher seroprevalence (23.6%). Titers of 800 or higher were found in 23 animals, most of which were from the municipality of Pacaraima. The Australis serogroup was the most common in this group. This study reports the first evidence of *Leptospira* exposure in Lavradeiro horses, with higher seroprevalence in flood-prone areas and reactivity to uncommon serovars. Our findings highlight pathogen circulation in equine under semi-feral conditions and the need for integrated surveillance and conservation of the Lavradeiro horse in the Northern Brazilian Amazon.

## Introduction

Leptospirosis is a globally distributed zoonosis caused by spirochetes of the genus *Leptospira*, affecting multiple domestic animals, wildlife, and humans, with important implications for public and veterinary health (WOAH 2024). Environmental persistence of the agent depends on reservoir hosts that chronically shed leptospires in urine, contaminating soil and water, particularly in humid or aquatic environments. Transmission occurs mainly through contact with contaminated water or soil via skin abrasions or mucous membranes, and vertical transmission has also been reported, with reproductive consequences (Adler and de la Peña Moctezuma 2010).

In equines, *Leptospira* infection is often subclinical, although clinical manifestations may include fever, lethargy, anemia, jaundice, renal failure, and equine recurrent uveitis, one of the most relevant clinical outcomes (Ellis 2015; Divers 2022). Infection during pregnancy may result in placentitis and late-term abortion, frequently without prior clinical signs (Di Azevedo and Lilenbaum 2022).

Serological surveys in Latin America demonstrate wide variability in equine seroprevalence, reflecting differences in climate, environment, and management (Pinto et al. 2017). In Northern Brazil, characterized by extensive management systems and high environmental humidity, elevated seroprevalence has been reported, with several serogroups circulating among horses (Ribeiro et al. 2018).

Lavradeiro horses are a naturally adapted equine ecotype that developed over centuries in the savannas of Roraima State, Northern Brazilian Amazon (Braga 2000). These horses live under semi-feral management within Indigenous communities, with minimal sanitary and nutritional intervention and frequent exposure to natural water sources and seasonal flooding (Braga 2019). Such conditions may favor *Leptospira* transmission and maintenance.

Considering the ecological and socioeconomic importance of Lavradeiro horses in the Amazon, as well as their close contact with Indigenous communities and the associated public health implications, studies on the dynamics of leptospirosis in this population remain scarce. Therefore, the objective of the present study was to determine the seroprevalence of anti-*Leptospira* antibodies in Lavradeiro horses from the state of Roraima, in the Brazilian Amazon, contributing epidemiological data to support surveillance, prevention, and control strategies in the region, as well as preservation of this naturally adapted unique ecotype.

## Materials and methods

### Study area and animals

**Fig. 1:**
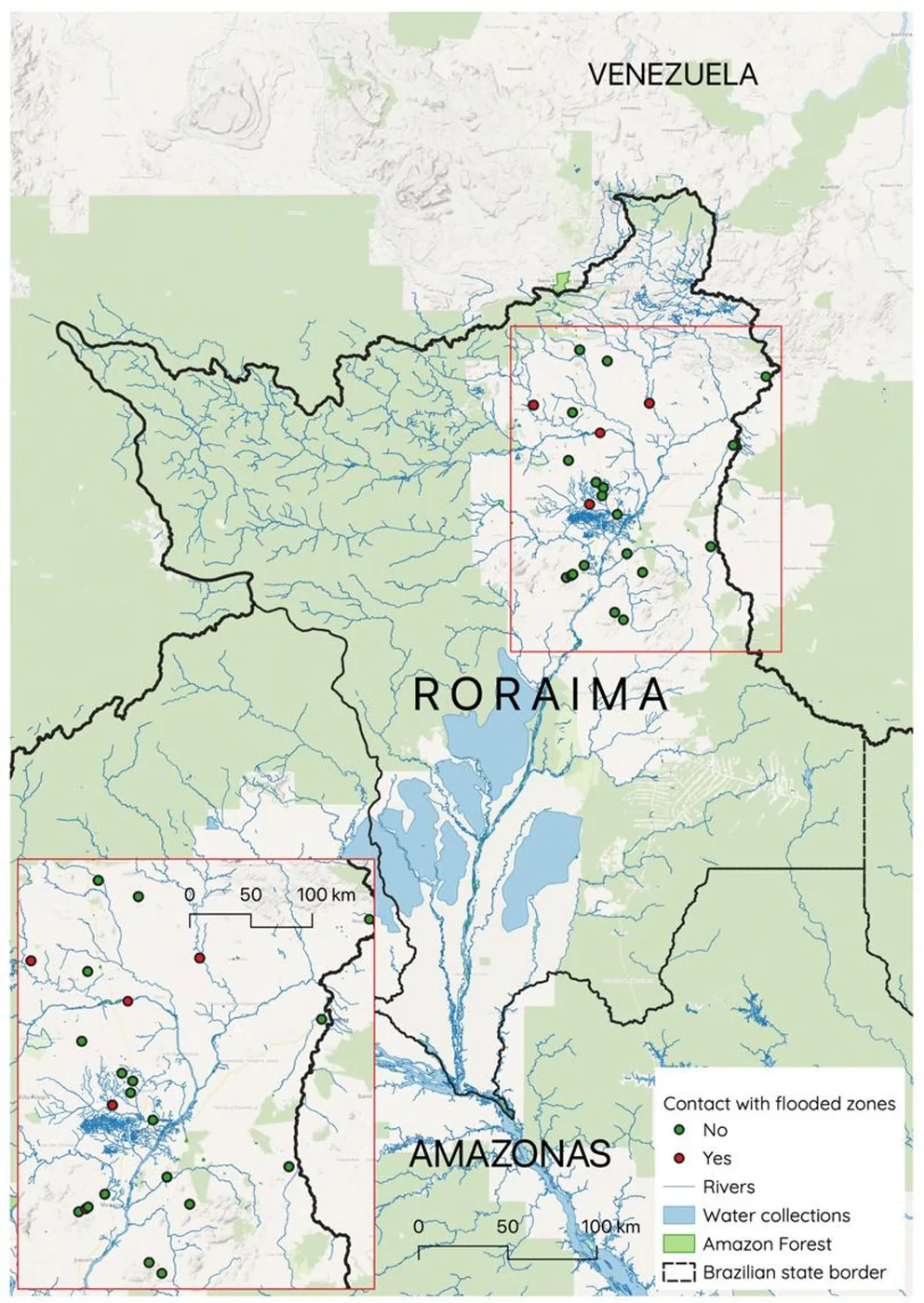
Location of sampled Indigenous communities according to reported contact with flooded zones in Roraima State, Brazil

Roraima is a state in northern Brazil located within the Legal Amazon, characterized by extensive areas of Amazon rainforest interspersed with savanna formations locally known as *lavrado*, which constitute the primary habitat of the Lavradeiro horse ecotype. The region presents a tropical savanna climate (Aw), with high temperatures and seasonal rainfall, conditions that favor the environmental persistence of *Leptospira* spp. Lavradeiro horses are raised under semi-feral conditions, with frequent exposure to natural water sources and flood-prone areas, which may increase the risk of leptospiral transmission (Fig. 1). According to data from the Brazilian Institute of Geography and Statistics (IBGE 2024), the national equine herd in 2024 comprised 5,702,612 animals, of which 42,191 were located in the state of Roraima, representing approximately 0.73% of the national total. These data refer to the overall equine population in the state rather than specifically to the Lavradeiro ecotype.

### Sample collection and statistical analysis

Samples were collected from Lavradeiro horses (*Equus caballus*) living under semi-feral management within Indigenous communities in the state of Roraima. The horses were randomly selected considering 95% confidence and 5% of significance Thrusfield 2007). The number of sampled horses in each municipality was proportional to the existing herd of each municipality. Moreover, the number of selected horses in each Indigenous community was proportional to the existing herd in each Indigenous community.

Due to these proportionality criteria, the seroprevalence of *Leptospira* spp. in Lavradeiro horses of the state of Roraima was calculated as the ratio of seropositive individuals to those tested, along with the corresponding 95% confidence interval, as described in Thrusfield (2007). These analyses were conducted utilizing the R software (R Core Team 2024).

The Lavradeiro horses were sampled from the following municipalities of Roraima state: Pacaraima, Boa Vista, Mucajaí, Amajari, Bonfim, Cantá, Alto Alegre and Normandia. Each animal was individually identified and categorised according to sex, age class (foals: 1–2 years; adults: 2–15 years; elderly: over 16 years), and geographical location, recorded using GPS coordinates. Blood was aseptically collected by jugular venipuncture and stored in sterile tubes. Blood samples were aseptically collected by jugular venipuncture into sterile tubes. The samples were maintained in a cooled container (approximately 4 °C) and processed within 3 h of collection. Processing consisted of centrifugation at 2,000 × *g* for 10 min to obtain serum. The sera were then transferred to 1.5 mL polypropylene microtubes, properly labeled, and stored at − 20 °C until serological analysis.

### Laboratory analysis

The Microscopic Agglutination Test (MAT) was performed according to WOAH (2024) and was executed by the Laboratory of Bacterial Zoonoses of the School of Veterinary Medicine of the University of São Paulo. A panel of 23 live *Leptospira* representing 18 serogroups was used (*Leptospira interrogans* serovars: Australis, Bataviae, Bratislava, Autumnalis, Canicola, Sentot, Hardjo type Prajitno, Hebdomadis, Copenhageni, Icterohaemorrhagiae, Pomona, Pyrogenes; *Leptospira borgpetersenii* serovars: Castellonis, Hardjo type Bovis, Javanica, Tarassovi and Whitcombi; *Leptospira noguchii* serovar: Panama; *Leptospira kirschneri* serovars: Butembo, Cynopteri and Grippotyphosa; *Leptospira santarosai* serovars: Guaricura and Shermani).

The screening of seroreactive animals utilized a 1:100 dilution. Reactive samples were subsequently examined with progressively increasing dilutions ranging from 1:100 to 1:3,200, with the highest positive dilution indicating the serum’s titer. A serum was deemed reactive when at least 50% agglutination was observed at a magnification of 100 times using a dark-field microscope (Guedes et al. 2021a). It should be noted that one of the limitations of MAT is its inability to distinguish between specific serovars; it is primarily used to identify serogroups. Therefore, the interpretation of serovar results should be approached with caution.

## Results

Serum samples from 387 Lavradeiro horses from Indigenous communities in the state of Roraima were analyzed. The Lavradeiro horses were sampled from the following municipalities of Roraima state: Pacaraima 24.3% (94/387), Boa Vista 16.5% (64/387), Mucajaí 14.2% (55/387), Amajari 13.7% (53/387), Bonfim 13.7% (53/387), Cantá 12.4% (48/387), Alto Alegre 4.9% (19/387) and Normandia 0.3% (1/387). Regarding sex, 178 were female, 197 were males, and 12 had no sex information. Regarding age category, 92 were foals, 278 adults, 4 elderly, and 13 had no age recorded.

The overall seroprevalence of *Leptospira* in all evaluated horses was 15.8% (61/387; 95% CI: 12.3–19.8%). The serogroups and serovars identified in seropositive animals are detailed in Table 1. The most frequent serogroups were Australis (Australis: 21%), Tarassovi (Tarassovi: 9%), Autumnalis (Autumnalis: 8%), Pomona (Pomona: 8%), and Djasiman (Sentot: 7%) (Table 1). The most likely infecting serovars, based on the highest titers and isolated reactivity, were Australis (16/56), Autumnalis (7/56), and Pomona (5/56).

**Table 1:** Frequency of *Leptospira* serogroups and serovars identified in seropositive Lavradeiro horses from Roraima, Brazil, and their respective highest antibody titers

Serogroup	Serovar	Horses (%)	Highest antibody titer
Djasiman	Sentot	7	800
Icterohaemorrhagiae	Icterohaemorrhagiae	3	1600
	Copenhageni	1	100
Australis	Bratislava	1	3200
	Australis	21	1600
Pomona	Pomona	8	2800
Ballum	Castellonis	3	400
Whitcombi	Whitcombi	0	-
Sjeroe	Hardjo type Bovis	0	-
	Hardjo type Pratino	3	400
	Guaricura	0	-
Autumnalis	Autumnalis	8	800
	Butembo	2	400
Canicola	Canicola	4	800
Gripphotyphosa	Gripphotyphosa	5	800
Tarassovi	Tarassovi	9	200
Shermani	Shermani	3	400
Cynopteri	Cynopteri	5	1600
Hebdomadis	Hebdomadis	0	-
Javanica	Javanica	0	-
Panama	Panama	1	100
Bataviae	Bataviae	1	800
Pyrogenes	Pyrogenes	0	-

When stratified by age category, seropositivity was observed in 17.6% of adult horses (49/278; 95% CI: 13.3–22.6) and in 9.8% of foals (9/92; 95% CI: 4.6–17.8). Elderly horses were not included in the statistical analysis due to their low number (*n* = 4). There was no statistically significant difference in seroprevalence between age groups (*p* = 0.073). Regarding sex, 16.8% of females (30/178; 95% CI: 11.7–23.2) and 14.7% of males (29/197; 95% CI: 10.1–20.4) were seropositive, with no significant difference between groups (*p* = 0.566).

Titers above 800 were detected in 23 animals, with a maximum titer of 3,200. Most of these cases occurred in horses from the municipality of Pacaraima (12/23), followed by Amajari (8/23), Boa Vista (2/23), and Bonfim (1/23) (Table 2). Among horses with high titers, the Australis serogroup (10/23) was the most prevalent, followed by Pomona (4/23), as shown in Table 2. Fifteen animals presented multiple titre reactions, and the detailed results are shown in Table 3.

**Table 2:** Distribution of Lavradeiro horses with high antibody titers (≥ 800) against *Leptospira* spp., according to municipality of origin and identified serogroups, Roraima, Brazil

Municipality	Number of animals	Identified serogroups
Pacaraima	12	Australis, Autumnalis, Bataviae, Djasiman, Gripphotyphosa
Amajari	8	Australis, Cynopteri, Canicola, Pomona, Icterohaemorrhagiae
Boa Vista	2	Australis
Bonfim	1	Australis
Total	23	-

**Table 3:** Distribution of Lavradeiro horses showing multiple serovar reactions to *Leptospira* spp., according to sex, age, municipality of origin, and identified serovars, Roraima, Brazil

Sex	Age	Municipality	Identified serovars and titers
female	foal	Amajari	Pomona (1600); Cynopteri (1600)
female	adult	Amajari	Australis (200); Shermani (200); Copenhageni (100)
female	adult	Amajari	Australis (200); Canicola (800); Sentot (200)
female	adult	Amajari	Bratislava (3200); Canicola (800); Tarassovi (100)
male	adult	Mucajai	Icterohaemorrhagiae (400); Shermani (400); Tarassovi (200)
male	adult	Bonfim	Australis (800); Tarassovi (200)
male	adult	Bonfim	Australis (400); Tarassovi (200)
female	adult	Cantá	Australis (100); Sentot (200)
female	adult	Pacaraima	Australis (400); Tarassovi (200); Castellonis (100)
male	adult	Pacaraima	Australis (400); Bratislava (800)
male	adult	Pacaraima	Australis (1600); Sentot (800)
male	adult	Pacaraima	Shermani (100); Sentot (200)
female	adult	Pacaraima	Australis (800); Gripphotyphosa (100)
female	adult	Pacaraima	Australis (800); Autumnalis (400); Tarassovi (100)
female	adult	Pacaraima	Australis (1600); Tarassovi (200); Sentot (400)

**Fig. 2:**
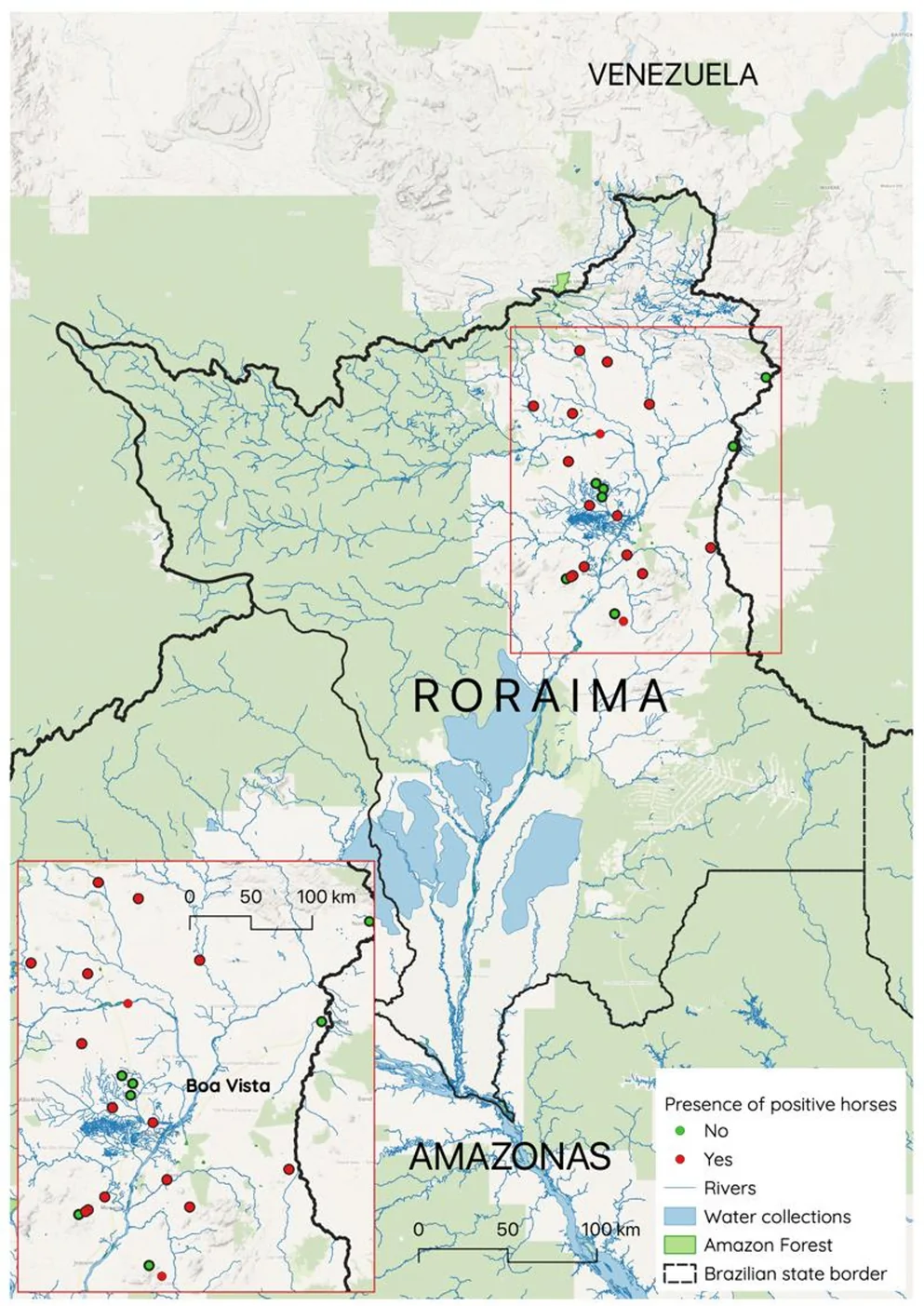
Distribution of sampled Indigenous communities according to the presence or absence of horses with anti-*Leptospira* antibodies in Roraima State, Brazil

Seroprevalence was also assessed in relation to flood-prone areas, based on geographic classification by IBGE. Horses from flood-susceptible areas had a higher serological prevalence (23.6%; 21/89; 95% CI: 5.2–33.8) than those from non-flooded areas (13.6%; 40/295; 95% CI: 9.9–18.0) (Fig. 2). This difference was statistically significant (*p* = 0.023), suggesting that environmental conditions may influence exposure to the etiological agent.

## Discussion

The serogroups most traditionally associated with leptospirosis in Brazilian equines include Icterohaemorrhagiae, Australis, and Pomona (Pinto et al. 2017). In the present study, the Australis serogroup was the most prevalent, identified in 21% of seropositive animals. Although the overall seroprevalence was relatively low (15.8%), the detection of high titers in some horses suggests possible active infection rather than mere exposure. Among animals with high titers, the Australis serovar was the most frequent (10/23), reinforcing its epidemiological importance in the region. Although the overall prevalence of 15.8% is not considered high, the occurrence of titers ≥ 800 in 23 animals suggests possible active infection in part of the population, rather than previous exposure. This condition is particularly relevant in Lavradeiro horses, especially considering their rusticity and frequent contact with natural environments favorable to *Leptospira* survival.

High seropositivity for Australis was expected, as this serogroup is considered adapted to the equine species (Ellis 2015). Nevertheless, it is noteworthy that only 1% of the animals seropositive for the Bratislava serovar, which belongs to the same serogroup and is commonly reported in horses. This finding may reflect local differences in serovar circulation or low infection pressure of Bratislava in the studied area, underscoring the need for further investigation.

The frequent detection of Autumnalis and Djasiman suggests prevalent circulation of these serogroups, potentially indicating transmission from wild animals (Fornazari et al. 2018). The Sentot serovar (Djasiman), for example, has been reported as prevalent in horses from the Amazon region (Ribeiro et al. 2018) and more recently in horses from the Central-West region of Brazil (Romanowski et al. 2023). Likewise, Autumnalis has been commonly found in equines, as observed in military horses in Chile (Tadich et al. 2016) and in studies conducted in Northern Brazil (Aguiar et al. 2008; Ulsenheimer et al. 2023). The Djasiman serovar was also recently identified as the most prevalent in equines from the Central-West region, with 16.4% positivity (Romanowski et al. 2023).

The presence of Pomona serovar in Brazilian horses has been documented, with prevalence varying by animal management (Romanowski et al. 2023). In the present study, Pomona was also detected among animals with high antibody titers (4/23), reinforcing its epidemiological relevance. This serovar is of particular concern due to its association with reproductive disorders in horses, although this aspect was not investigated in the present study as the semi-feral management conditions precluded such evaluation. Additionally, Pomona has been detected in wild suids, which may represent a source of infection for other animal species (Fornazari et al. 2011), potentially contributing to the exposure of horses to this serovar.

Although the frequency of the Icterohaemorrhagiae serovar was low in this study, its detection is relevant given its association with most human leptospirosis cases and its high prevalence in horses across Brazil (Martins and Lilenbaum 2013; Oliveira et al. 2017; Romanowski et al. 2023). Detection of antibodies against the Cynopteri serovar is uncommon in equines but was recorded in this study. This serovar has been identified in multiple mammalian hosts, including wild felids, primates, and horses from a mixed-use rescue center in Ecuador (Orlando et al. 2020). Additionally, Cynopteri has also been reported in bats in Argentina (Saraullo et al. 2021), supporting its circulation among different wildlife species. These findings suggest that exposure may occur in environments where domestic and wild animals coexist. Including this serovar in serological panels may therefore be relevant in regions with habitat overlap between wildlife and horses, although further studies are needed to clarify its epidemiological role.

The observed frequency of the Tarassovi serovar (9%) is also notable. Previous studies have reported its presence in various species, including cattle, deer, sheep, and horses, particularly in New Zealand, but also in cattle in the Amazon region of Brazil (Wilson et al. 2020; Guedes et al. 2021b). It is important to consider that MAT results may also reflect multiple exposures over time. Although simultaneous infections are considered less frequent, sequential exposure to different serovars cannot be ruled out, especially under natural conditions. Thus, it is not possible to determine whether reactions are due to a single infecting serovar or to successive exposures, which is a recognized limitation in epidemiological studies (Ellis 2015; Guedes et al. 2021a).

In addition, paradoxical reactions must be considered when interpreting MAT results. During acute infection, the predominance of IgM antibodies may result in equal or even higher titers against heterologous serovars compared to the infecting one, making interpretation difficult (Ellis 2015). Although such cross-reactions are classically associated with early infection, they may also be observed in epidemiological studies where the stage of infection is unknown due to random sampling (Guedes et al. 2021a). In this context, the absence of paired samples limits the ability to distinguish between acute and convalescent responses, and results should therefore be interpreted with caution.

Several studies indicate that freshwater plays a crucial role in the transmission of pathogenic *Leptospira* spp., and the risk of infection increases during floods (Amilasan et al. 2012) or in water-related recreational and occupational activities (Monahan et al. 2009). Experimentally, the viability and virulence of *Leptospira* spp. were maintained for at least 20 months under conditions mimicking tropical or temperate climates (Andre-Fontaine et al. 2015). The geographic distribution of Lavradeiro horses with high antibody titers (> 1:800) showed that most cases occurred in the municipality of Pacaraima, followed by Amajari, Boa Vista, and Bonfim. The presence of animals in flood-prone areas, common in these locations, supports the association between flooding and increased risk of leptospiral infection in Lavradeiro horses. The detection of high antibody titers highlights the need for effective leptospirosis surveillance and control strategies, especially considering the role of horses as environmental sentinels that can reflect the presence and circulation of *Leptospira* in local ecosystems.

Equids may become infected with *Leptospira* spp. through contact with contaminated water or feed or via direct exposure to the urine or reproductive fluids of infected animals. Various wildlife species can act as asymptomatic carriers, shedding the bacteria into the environment and thereby serving as reservoirs of infection. Under semi-feral conditions, Lavradeiro horses are particularly at risk due to their proximity and interaction with wildlife, as well as frequent exposure to natural water sources and seasonal flooding, which facilitate the maintenance and transmission of the pathogen.

Furthermore, MAT has important limitations in detecting chronic infection in individual animals. Chronically infected animals may present low or even undetectable antibody titers, sometimes below the commonly accepted cut-off, leading to false-negative results. According to the World Organisation for Animal Health, such animals may remain carriers and sources of infection despite negative serology. Therefore, MAT is more appropriately interpreted as a herd-level test, and its results should be considered alongside epidemiological context, particularly in cross-sectional studies such as the present one (WOAH 2024).

## Conclusion

In conclusion, our study provides the first serological evidence of *Leptospira* exposure in Lavradeiro horses, with higher seroprevalence observed in flood-prone areas, suggesting a possible environmental influence on transmission in the Roraima savanna. Reactivity to less commonly reported serovars indicates the potential ecological complexity of leptospirosis in tropical environments and highlights the need for further region-specific investigations. These findings contribute to the understanding of pathogen exposure among equids under semi-feral conditions may support future surveillance efforts within Indigenous territories of the northern Brazilian Amazon, alongside strategies for preservation of the Lavradeiro ecotype.

## Data Availability

Data will be made available upon reasonable request.

## References

[CR1] Adler B, de la Peña Moctezuma A (2010) Leptospira and leptospirosis. Vet Microbiol 140:287–296 19345023 10.1016/j.vetmic.2009.03.012

[CR2] Aguiar DM, Moraes LM, Oliveira DS et al (2008) Prevalência de anticorpos contra agentes virais e bacterianos em eqüídeos do Município de Monte Negro, Rondônia, Amazônia Ocidental Brasileira. Braz J Vet Res Anim Sci 45:269–276. 10.11606/issn.1678-4456.bjvras.2008.26685

[CR3] Amilasan AS, Ujiie M, Suzuki M et al (2012) Outbreak of leptospirosis after flood, the Philippines, 2009. Emerg Infect Dis 18:91–94. 10.3201/eid1801.101892 22257492 PMC3310081

[CR4] Andre-Fontaine G, Aviat F, Thorin C (2015) Waterborne leptospirosis: Survival and preservation of the virulence of pathogenic *Leptospira* spp. in fresh water. Curr Microbiol 71:136–142. 10.1007/s00284-015-0836-4 26003629

[CR5] Braga RM (2000) Cavalo lavradeiro em Roraima – Aspectos históricos, ecológicos e de conservação. 1ª ed. Embrapa, Brasília, Brasil, v. 96p

[CR6] Braga RM (2019) Cavalo Lavradeiro: aspectos históricos, situação atual, desafios e possíveis soluções para sua conservação. Embrapa Roraima, Boa Vista, Brasil

[CR7] Di Azevedo MIN, Lilenbaum W (2022) Equine genital leptospirosis: Evidence of an important silent chronic reproductive syndrome. Theriogenology 192:81–88. 10.1016/j.theriogenology.2022.08.029 36063673

[CR8] Divers TJ (2022) Acute kidney injury and renal failure in horses. Vet Clin Equine Pract 38:13–24. 10.1016/j.cveq.2021.11.002 35282961

[CR9] Ellis WA (2015) Animal leptospirosis. In: Adler B (ed) Leptospira and Leptospirosis. Springer, Berlin/Heidelberg, pp 99–137

[CR10] Fornazari F, Camossi LG, Silva RC et al (2011) Leptospiral antibodies in wild boars (*Sus scrofa*) bred in Brazil. J Venom Anim Toxins Incl Trop Dis 17:94–97. 10.1590/S1678-91992011000100012

[CR11] Fornazari F, Langoni H, Marson PM et al (2018) *Leptospira* reservoirs among wildlife in Brazil: Beyond rodents. Acta Trop 178:205–212. 10.1016/j.actatropica.2017.11.019 29197499

[CR12] Guedes IB, Souza GO, Castro JFP et al (2021a) Usefulness of the ranking technique in the microscopic agglutination test (MAT) to predict the most likely infecting serogroup of *Leptospira*. Front Vet Sci 8:636382. 10.3389/fvets.2021.654034 PMC7965942 33748224

[CR13] Guedes IB, Souza GO, Rocha KS et al (2021b) *Leptospira* strains isolated from cattle in the Amazon region, Brazil: Evidence of a variety of species and serogroups with a high frequency of the Sejroe serogroup. Comp Immunol Microbiol Infect Dis 74:101579. 10.1016/j.cimid.2020.101579 33246243

[CR14] IBGE (2024) — Instituto Brasileiro de Geografia e Estatística. Produção Agropecuária | Equinos. https://www.ibge.gov.br/explica/producao-agropecuaria/equinos/br. Accessed 3 Nov 2025

[CR15] Martins G, Lilenbaum W (2013) The panorama of animal leptospirosis in Rio de Janeiro, Brazil, regarding the seroepidemiology of the infection in tropical regions. BMC Vet Res 9:1–7. 10.1186/1746-6148-9-237 24289165 PMC4220826

[CR16] Monahan AM, Miller IS, Nally JE (2009) Leptospirosis: risks during recreational activities. J Appl Microbiol 107:707–716. 10.1111/j.1365-2672.2009.04220.x 19302325

[CR17] Oliveira MAA, Leal EA, Correia et al (2017) Human leptospirosis: Occurrence of serovars of *Leptospira* spp. in the state of Minas Gerais, Brazil, from 2008 to 2012. Braz J Microbiol 48:483–488. 10.1016/j.bjm.2016.12.010 28365095 PMC5498453

[CR18] Orlando SA, Perez A, Sanchez E et al (2020) High seroprevalence of anti-*Leptospira* spp. antibodies in domestic and wild mammals from a mixed use rescue center in Ecuador: Lessons for One Health based conservation strategies. One Health 10:100140. 10.1016/j.onehlt.2020.100140 32426447 PMC7226863

[CR19] Pinto PS, Libonati H, Lilenbaum W (2017) A systematic review of leptospirosis on dogs, pigs, and horses in Latin America. Trop Anim Health Prod 49:231–238. 10.1007/s11250-016-1201-8 27909915

[CR20] R Core Team (2024) R: A language and environment for statistical computing. R foundation for statistical computing, Vienna, Austria. Available in: www.R-project.org

[CR21] Ribeiro TMP, Correia L, Spohr KAH et al (2018) Risk factors associated with seroreactivity against *Leptospira* sp. in horses from Brazilian Amazon. J Equine Vet Sci 68:59–62. 10.1016/j.jevs.2018.05.197 31256890

[CR22] Romanowski TNA, Dias RA, Heinemann MB et al (2023) Seroprevalence of equine leptospirosis in the State of Goiás, Brazil. Vet Sci 10:590. 10.3390/vetsci10100590 37888542 PMC10610622

[CR23] Saraullo V, Loffler SG, Pastorino F et al (2021) First report of pathogenic *Leptospira* spp. in *Tadarida brasiliensis* bats (family Molossidae) and *Eptesicus furinalis* (family Vespertilionidae) of Argentina. New host species in this country? Rev Argent Microbiol 53(3):210–215. 10.1016/j.ram.2020.09.007 33468355

[CR24] Tadich TA, Tapia C, González D (2016) Seroprevalence of *Leptospira* spp. in working horses located in the central region of Chile. J Equine Vet Sci 38:14–18. 10.1016/j.jevs.2015.12.011

[CR25] Thrusfield M (2007) Veterinary epidemiology, 3rd edn. Blackwell Science Ltd. pp, London, pp 275–290

[CR26] Ulsenheimer BC, Barboza CL et al (2023) Seroepidemiology of leptospirosis in horses from Santarém, Pará. Cienc Anim Bras 24. 10.1590/1809-6891v24e-74800E. e-74800P

[CR27] Wilson P, Mannewald A, Collins-Emerson J et al (2020) Serological study of *Leptospira interrogans* serovar Copenhageni and *L. borgpetersenii* serovars Tarassovi and Ballum in beef cattle, sheep and deer in New Zealand. N Z Vet J 69(2):83–92. 10.1080/00480169.2020.1830867 33183158

[CR28] World Organization for Animal Health (WOAH) (2024) Manual of diagnostic tests and vaccines for terrestrial animals. 13th ed. WOAH, Paris, France. Chapter 3.1.12: Leptospirosis

